# An Intensive Care Unit Outbreak of Acute Respiratory Distress Syndrome due to Human Metapneumo Virus Infection

**DOI:** 10.1177/2324709619860549

**Published:** 2019-07-04

**Authors:** Ankur Sinha, Vignesh Ponnusamy, Sushilkumar S. Gupta, Hitesh Raheja, Ravikaran Patti, Parita Soni, Namrita Malhan, Yu Shia Lin, Yizhak Kupfer

**Affiliations:** 1 Maimonides Medical Center, Brooklyn, NY, USA

**Keywords:** acute respiratory distress syndrome, metapneumo virus, intensive care unit, extracorporeal membrane oxygenation

## Abstract

Human metapneumo virus is an emerging cause of upper and lower respiratory tract illness with increasing reports of a varied spectrum of disease over all age groups. We report an outbreak of 6 cases of human metapneumo virus infection in the intensive care unit of a metropolitan tertiary care center over 6 weeks, leading to severe acute respiratory distress syndrome. We report the subsequent favorable outcomes due to the institution of extracorporeal membrane oxygenation.

## Introduction

Human metapneumo virus (hMPV) is an emerging cause of upper and lower respiratory tract illness. Since its initial isolation from the nasopharynx of children in 2001, there have been significant gaps in our understanding of its virulence and clinical features. Vigilant reporting of findings is warranted to aid physicians in managing patients with this infection. We report our findings while managing an outbreak of hMPV in the medical intensive care unit (ICU) of a metropolitan referral center with 6 cases of acute respiratory distress syndrome (ARDS) over a period of 6 weeks.

## Case Report

A retrospective review of 6 patients with confirmed hMPV over the period of 6 weeks was carried out. Inclusion criteria were clinical evidence of respiratory failure and a respiratory viral panel confirming metapneumo virus infection. The patient parameters including demographics, initial presentation, comorbidities, radiological findings, disease severity, ICU stay, and outcomes are documented in Table 1.

**Table 1.** Patient Characteristics and Outcomes.

Patient	Age (Years)/Sex	Presentation	Comorbidities	Chest Radiography	Onset of ARDS	Blood Culture	Respiratory Smear	Intubation	ECMO	Length of ICU Stay	Outcome
1	82/female	Cough, SOB, hypoxia	DM, HTN, pericarditis with window, breast cancer, aortic stenosis, UTI	Diffuse bilateral infiltrates	Day 3	Negative	*Staphylococcus aureus*	No—DNR DNI	No	19 days	Deceased
2	67/female	Cough with sputum, SOB, fever, chills, myalgia	HTN, hypothyroidism, hiatus hernia	Diffuse bilateral infiltrates	Day 1	Negative	*Streptococcus pneumoniae*	Yes	Yes	22 days	Survived
3	51/female	Cough with sputum, SOB, fever, chills	DM, HTN, psittacosis, former smoker	Diffuse bilateral infiltrates	Day 1	Negative	*Streptococcus pneumoniae*	No	No	4 days	Survived
4	58/female	Cough with sputum, SOB, fever	DM, HTN, CVA	Diffuse bilateral infiltrates	Day 1	Coagualse negative *Staphylococcus*	Negative	No	No	9 days	Survived
5	18/female	Cough, fever, nausea, vomiting, premature contractions	Pregnancy	Diffuse bilateral infiltrates	Day 3	Negative	Negative	Yes	Yes	8 days	Survived
6	35/female	Cough, AMS, diarrhea, vomiting, fever, chills	None	Diffuse bilateral infiltrates	Day 1	Negative	Negative	Yes	Yes	14 days	Survived

Abbreviations: ARDS, acute respiratory distress syndrome; ECMO, extracorporeal membrane oxygenation; ICU, intensive care unit; SOB, shortness of breath; DM, diabetes mellitus; HTN, hypertension; UTI, urinary tract infection; DNR, do not resuscitate; DNI, do not intubate; CVA, cerebrovascular accident; AMS, altered mental status.

All the patients in our study presented from the community and the infection was presumed to be community acquired. They were presumed to have a viral infection on admission, and droplet isolation precautions in addition to regular universal precautions were employed as per hospital infection control policy. The age range was 18 to 82 years. Although all the patients in our study were female, it is not possible to comment on a correlation due to a relatively small sample size. Cough was the most consistent clinical feature at presentation and was seen in all the individuals; only 50% (3 patients) of these individuals reported sputum production with this cough. Shortness of breath was another consistent finding (66%, 4 patients) as well as fever with chills (66%, 4 patients). Two patients had severe nausea and vomiting. One of these patients was pregnant and had premature contractions.

The commonest radiographic finding was a diffuse bilateral infiltrate on a portable anteroposterior view radiograph of the chest. All of our patients met criteria for ARDS as per the revised Berlin definition, on account of acute onset, diffuse infiltrates not explained by any other cause, and hypoxia. The onset of ARDS was on day 1 for 66% (4 patients) of the patients and day 3 for 33% (2 patients).

A respiratory culture and smear was performed for all patients in addition to a rapid viral panel on the day of presentation. All the patients had a positive viral panel for hMPV, whereas 3 patients had a positive respiratory culture, with *Staphylococcus aureus* in 1 patient and *Streptococcus pneumoniae* in the other 2 patients. Blood culture was positive for coagulase-negative *Staphylococcus* in 1 patient, although this patient had a negative respiratory culture.

Out of these 6 patients with ARDS, 66% (4 patients) needed intubation. One of the patient’s family refused intubation and she did not survive the disease. All of the 3 intubated patients were placed in prone position based on the PaO_2_/FiO_2_ ratios and eventually needed extracorporeal membrane oxygenation (ECMO). They were started on a venovenous ECMO and transferred to the cardiothoracic ICU. They were successfully weaned off the ECMO. One of the patients needed a tracheostomy as she could not be successfully weaned off mechanical ventilation. The 2 patients who did not require intubation were treated with high-flow oxygen via nasal prongs and improved without requiring invasive ventilation. All 5 patients who survived the disease continued to do well on a 1-year follow-up.

## Discussion

hMPV is a ribonucleic acid virus with a single strand of negative sense, non-segmented ribonucleic acid. It is classified in the Paramyxoviridiae family, Pneumovirinae subfamily, *Metapneumovirus* genus.^1^ It belongs to the same subfamily as the human respiratory syncytial virus.

hMPV was first described in 2001, and as per some surveys of prevalence in the Netherlands, virtually all children are infected by the age of 5 to 10 years.^2^ This information along with several cases of hMPV in adults points toward an incomplete immune response. hMPV infection has a seasonal distribution, with studies reporting distribution over winter and early spring months.^3^ The peak incidence was found to be in early spring, slightly later than the outbreak period of the respiratory syncytial virus.^4^ hMPV circulation follows a seasonal pattern with a biennial distribution from November to February and then from April to July.^5^ Our findings corroborated with these observations as all the cases in our study presented in the months of November and December.

The clinical profile of hMPV infection varies greatly and is affected by factors like age, the presence of comorbid conditions, co-infection with another pathogen, and immune suppression. Majority of hMPV infection are asymptomatic, with studies indicating that as high as 38% of hMPV infections in nonhospitalized adults are asymptomatic.^6^ The spectrum of the disease involves upper as well as lower respiratory tract infection. hMPV was found to cause approximately 4% of all cases of community-acquired pneumonia as per a prospective cohort study, and these findings were confirmed by a similar study.^7,8^

Based on histopathological studies conducted on lung tissue obtained from cases of severe hMPV pneumonia, clinically distinct smudge cells with adjoining areas of severe organizing injury were identified. These findings differentiate hMPV from other paramyxoviral infections.^9^ Experimental animal model studies have aided in our understanding of hMPV pathogenesis. Interleukins (IL), namely IL-2, IL-4, IL-8, and IL-10, and interferons (IFN), IFN-γ and IFN-α, were identified as the main mediators in mice and cotton rats following infection with hMPV.^10^

Severe disease results in patients with coexisting conditions. Patients with hematological malignancies and recipients of human stem cell transplant had a higher incidence of symptomatic disease. Human stem cell transplant recipients had high mortality associated with symptomatic disease with studies reporting mortality numbers as high as 80%.^11^ A randomized controlled study performed in children showed that co-infection with *Streptococcus pneumoniae* may also lead to significant morbidity and increased rate of hospitalization. This conclusion was based on better outcomes of hMPV infection in children who had received 3 doses of pneumococcal vaccine instead of placebo.^12^

hMPV infection can be detected from sputum/respiratory secretions using reverse transcriptase polymerase chain reaction. Reverse transcriptase polymerase chain reaction has been universally accepted as the most reliable method of diagnosis with the highest sensitivity.^13,14^ Immuno-chromatography based rapid antigen tests have been developed as well. Our institution incorporated this test as a part of a rapid viral panel, which aided in diagnosing our patients with hMPV. It has a sensitivity of 82.3% and a specificity of 93.8%.^15^

The treatment of hMPV is vigilant supportive measures to maintain adequate hydration. The patient should have a chest radiograph for visualization as well as for establishing a baseline. The presence of infiltrates, as well as bilateral lung involvement, should alert the physician to the possibility of impending respiratory failure. Strict monitoring of patients with bilateral lung infiltrates should be conducted with several parameters like blood pressure, oxyhemoglobin saturation, and arterial blood gases. The patient should ideally be placed in the medical ICU.

hMPV can cause acute hypoxemic respiratory failure, and prompt endotracheal intubation with mechanical ventilation can be life-saving. Patients with confirmed ARDS requiring ventilation should be supported with low-volume ventilation to maintain oxygenation while avoiding lung injury. The Acute Respiratory Distress Syndrome Network (ARDSnet) studied the difference in clinical outcomes between traditional tidal volumes of 12 mL per kg of ideal body weight versus a lower tidal volume (6 mL/kg of ideal body weight). The trial was stopped midway because the mortality was found to be significantly higher in the higher tidal volume group (39.8% vs a lower 31% mortality in the lower tidal volume group).^16^

Blood gas parameters, especially the partial pressure of dissolved oxygen in the blood (PaO2) are an important marker of gas exchange. The PaO_2_/FiO_2_—the ratio of arterial oxygen tension to the fraction of inspired oxygen—is an important parameter in determining the course of treatment. As discussed above, the PaO_2_/FiO_2_ is used to gauge the severity of ARDS, and values less than 150 denote severe ARDS.

Based on the severity of ARDS, clinicians should consider placing the patient in prone position, as well as instituting a neuromuscular blocking agent. Studies have shown that prone positioning improves aeration as well as strain distribution across lung fields thereby improving oxygenation.^17^ Prone positioning should be initiated within 48 hours of the advent of ARDS. The best clinical outcome is achieved when a patient is placed in prone position with concurrent use of low tidal volume ventilation as well as neuromuscular blockade.

**Figure 1.**
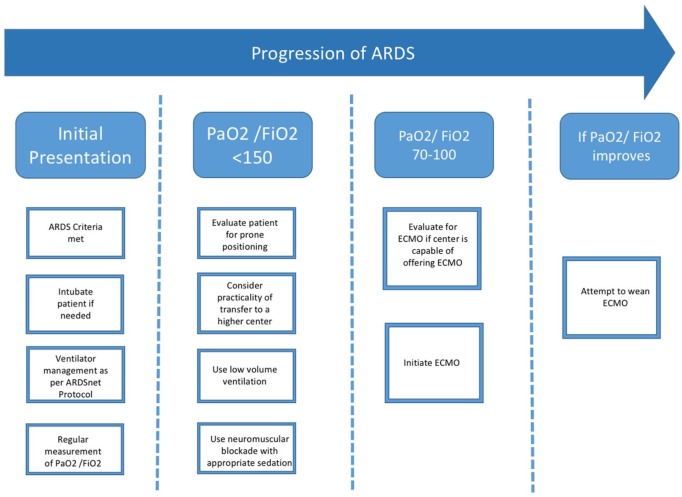
Schema depicting our treatment strategy for patients with ARDS (ARDS, acute respiratory distress syndrome; ECMO, extracorporeal membrane oxygenation).

While the patient is maintained on the above-mentioned parameters, clinicians should consider the early involvement of multidisciplinary teams that can help offer ECMO to the patient if needed. The Berlin consensus recommended instituting ECMO if the PaO_2_/FiO_2_ ratio is less than 70.^18^ The Conventional ventilator support versus Extracorporeal membrane oxygenation for Severe Acute Respiratory failure trial compared outcomes of management with conventional mechanical ventilation versus referral to a higher center with facilities for performing ECMO. The group of patients that were referred to the higher centers had increased survival without disability after 6 months (63% in the referred group vs 47% in the conventional group).^19^ Based on these recommendations coupled with evidence of severe ARDS in our patients, we initiated ECMO in our patients, and they were successfully weaned. Figure 1 depicts our treatment strategy while caring for our patients with ARDS.

## Conclusions

With a rapid advent in the prevalence of hMPV infection and its potentially life-threatening course, it is prudent to consider hMPV among the differentials of rapidly progressive respiratory illnesses. Timely transfer to the ICU with regular monitoring for ARDS and prompt interventions can prove life-saving.
